# Macroevolution of protective coloration across caterpillars reflects relationships with host plants

**DOI:** 10.1098/rspb.2022.2293

**Published:** 2023-01-25

**Authors:** Moria L. Robinson, Marjorie G. Weber, Micah G. Freedman, Evan Jordan, Sarah R. Ashlock, Jenna Yonenaga, Sharon Y. Strauss

**Affiliations:** ^1^ Center for Population Biology, University of California, Davis, CA 95616, USA; ^2^ Department of Biology, Utah State University, Logan, Utah 84322, USA; ^3^ Department of Ecology and Evolutionary Biology, University of Michigan, Ann Arbor, MI 48109, USA; ^4^ Department of Ecology and Evolution, University of Chicago, Chicago, IL 60637, USA; ^5^ Wissenschaftskolleg zu Berlin, Berlin, 14193, Germany; ^6^ Department of Evolution and Ecology, University of California, CA 95616, USA

**Keywords:** protective coloration, plant defence theory, plant–herbivore coevolution, tri-trophic interactions, warning coloration, camouflage

## Abstract

A critical function of animal coloration is avoiding attack, either by warning predators or reducing detectability. Evolution of these divergent strategies may depend on prey palatability and apparency to predators: conspicuous coloration may be favoured if species are distasteful, or habitats make hiding difficult; by contrast, camouflage may be effective if prey lack defences or environments are visually complex. For insect herbivores, host plants provide both chemical defence and the background against which they are detected or obscured; thus, plant traits may be key to coloration in these foundational terrestrial organisms. We use 1808 species of larval Lepidoptera to explore macroevolution of protective coloration strategy. We find that colour and pattern evolve jointly in caterpillars, similar to an array of species across the animal kingdom, while individual elements of coloration evolve closely with diet ecology. Consistent with key tenets of plant defence and plant–herbivore coevolutionary theory, conspicuous colours are associated with herbaceous host plants—thought to be defended by toxins—while camouflage colours and patterns are associated with woody plants and grasses. Contrary to theory, dietary specialization is not associated with conspicuous coloration. Our results add valuable insights into the evolutionary forces shaping colour and pattern in nature.

## 1. Introduction

The dazzling array of coloration in the animal kingdom is one of the most captivating aspects of the natural world. For prey, certain colours and patterns can signal danger or inedibility to predators, while others allow them to escape detection [1]. Understanding the factors that favour the evolution of one strategy over the other has puzzled biologists for centuries: Darwin was baffled by conspicuous caterpillars, as their bright colours could not be used to signal to mates yet should increase predation risk [2]. Wallace suggested that colourful caterpillars were probably toxic, and that predators should only eat dull, camouflaged ones [2]. While Wallace's ideas have been borne out in many systems [3], recent reviews highlight a need for broad-scale, hypothesis-driven studies to understand the ecological factors that shape evolution of protective coloration strategy [1]. On the one hand, theory predicts that anti-predator coloration should reflect the defensive ability of prey: those that are unpalatable to predators should evolve conspicuous coloration, while palatable species should evolve to blend in. On the other hand, the visual background against which species are detected or obscured is also theorized to impact colour evolution, with habitats or behaviours that make prey unavoidably apparent favouring evolution of conspicuous coloration, and visually complex signal environments selecting for phenotypes that minimize detection [4–6]. Together, these ideas provide a theoretical framework to understand when conspicuous versus camouflage coloration might evolve.

Across diverse taxa, certain colours, patterns and their combinations are thought to confer warning or hiding functions. For example, conspicuous, high-contrast colours, often in association with symmetrical, repeating patterns have been found to deter predators of many prey species: transverse bands of black with white, red, orange or yellow signal toxicity in snakes [7] and millipedes [8]; and toxic amphibians sport round spots and conspicuous colours [9]. Alternatively, colours such as green, brown and grey [10], often in combination with irregular blotches are thought to camouflage mammals [11], reptiles [10,12] and even plants [13] within their vegetated habitats. Hiding may also be conferred by longitudinal stripes, aligning with linear grassy habitats [14] and twig or stem perches [12,15]; and tiny ‘stippled’ dots that countershade prey and reduce dimensionality [16].

**Figure 1.**
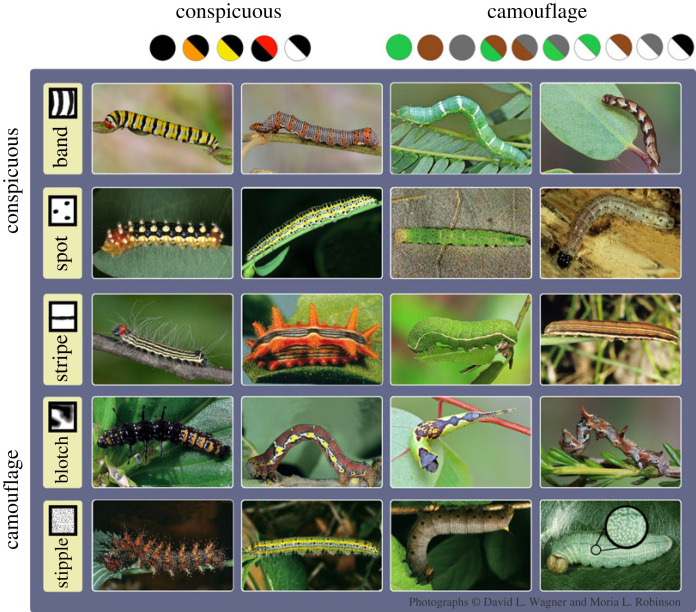
Caterpillars exhibit a large range of pattern and colour combinations, which together confer conspicuousness or camouflage as visual defences (table 1). Previous studies have shown that band and spot patterns can be part of warning coloration, blotches and stippling can be associated with camouflage and stripes can be effective for both strategies. Red, white, yellow and orange colours in combination with black are also often associated with conspicuous warning coloration, while background-matching colours—green, brown, grey and their combinations with white are associated with camouflage that reduces prey detection (table 1; electronic supplementary material, S1). Here, we illustrate the coloration diversity found in North American caterpillars, and how they were scored for pattern and colour traits. Top row, band: lines entirely or mostly encircling the body. Second row, spot: symmetrical round shape. Third row, stripe: longitudinal line(s) running the length of the body. Fourth row, blotch: irregular shape, often does not repeat. Fifth row, stipple: many tiny dots (see enlargement). Many species contain multiple pattern elements; for example, *Eudryas grata* (first row, second column) would be scored band, spot; *Ascia monuste* (fifth row, second column) would be scored as stipple, spot and stripe. See the electronic supplementary material for all species. Photographs © David L. Wagner and Moria L. Robinson.

Here, we capitalize on the unique biology of larval Lepidoptera (caterpillars) to explore the evolution of colour and pattern, and test for predicted signatures of trophic interactions on the evolution of protective coloration across the caterpillar phylogeny. Caterpillars are powerful subjects for this question because they display an impressive array of coloration (figure 1), are coveted prey of visual predators [17] and do not signal to mates. Furthermore, owing to metamorphosis, larval and adult coloration are largely independent [18]. Thus, selection on caterpillar coloration from predators can be separated from sexual selection—which can otherwise confound studies of protective coloration [19].

**Table 1.** Protective coloration and its relationship with ecology across the animal kingdom. (In a broad array of taxa, certain colours are often found with certain patterns, conferring conspicuousness and camouflage (A,B). These colour-pattern ‘syndromes’, or individual colours or patterns within them, are often found in association with diet or habitat (C–K). We use this literature to form hypotheses of associations between colour, pattern and diet ecology in caterpillars, and categorize models testing these hypotheses as exploratory or *a priori* (see the electronic supplementary material, text and table S2 for more details, and for full reference list).)

commonalities in protective coloration across a large diversity of taxa		
association among traits	found in…	references
**A.** *conspicuous coloration*—black with yellow, orange, red, white; high-contrast, regular patterns of bands (transverse lines), stripes (longitudinal lines) or spots	snakes, millipedes, caterpillars, model prey of birds and dragonflies, skunks, ladybirds and frogs	Brodie [20]; Marek & Bond [8]; Berenbaum [21] ; Smith [22]; Barnett *et al*. [23]; Kauppinen & Mappes [24]; Hunter [25]; Arenas *et al*. [26]; Preißler & Pröhl [9]
**B.** *camouflage coloration*—green, brown, grey; irregular shapes (blotch), countershading via tiny dots (stippling), stripes to conceal against linear vegetation forms	felids, snakes, butterfly chrysalids, caterpillars, *Timema* stick insects, plants	Allen *et al*. [11]; Allen *et al*. [10]; Poulton [16]; Beddard [27]; Heinrich [28]; Sherman & Watt [15]; Mariath [29]; Sandoval [30]; Givnish [13]
**C.** conspicuous coloration associated with toxic diets, consumption of N-containing compounds	poison frogs, Heteroptera, *Platyphora* leaf beetles, marine opisthobranchs; caterpillars; aphids	Santos *et al*. [31]; Aldrish [32]; Termonia *et al*. [33]; Cortesi & Cheney [34]; Nishida [35]; Opitz & Muller [36]
**D.** conspicuous coloration associated with simple microhabitats; camouflage with complex ones	*Papilio* caterpillars, terrestrial carnivores, theoretical models	Prudic [5]; Dimitrova & Merilaita [37]; Stankowich *et al*. [38]; Higginson *et al*. [39]
**E.** grass diets or habitats associated with stripes; camouflage colours	caterpillars, bittern (bird) in reeds, tiger in long grass, geckos	Beddard [27]; Godfrey *et al*. [14]; Allen *et al*. [12]
**F.** woody plant diets or habitats associated with camouflage coloration	felids, snakes, *Papilio* caterpillars	Allen *et al*. [11]; Allen *et al*. [10]; Prudic *et al*. [5]
**G.** specialization associated with either conspicuousness or camouflage	poison frogs, spotted lanternfly, caterpillars, *Timema* stick insects, grasshoppers	Santos *et al*. [31] ; Song *et al*. [40]; Nishida [35]; Bernays & Cornelius [41]; Sandoval [30]; Otte & Joern [42]
**H.** generalization associated (commonly) with camouflage but (possibly) with conspicuousness	poison frogs, spotted lanternfly, theoretical models	Santos *et al*. [31]; Song *et al*. [40]; Nishida [35]; Merilaita & Tullberg [43]
**I.** concealed feeding or burrowing associated with few visual signals; lack of colours/patterns	burrowing mammals, caecilians, caterpillars	Stankowich *et al*. [38]; Wollenberg & Measey [44]; Dyar [45]; Le Rü *et al*. [46]
**J.** florivory associated with conspicuous coloration	theoretical models, caterpillars	Higginson *et al*. [39]; Morais *et al*. [47]; McCall & Irwin [48]
**K.** detritus, fungi, or lichen diets or microhabitats associated with camouflage coloration	snakes, *Phrynosoma* horned lizards, forest viper, frogs	Allen *et al*. [10]; Sherbrooke [49]; Branch & Bayliss [50]; Ferreira *et al*. [51]

First, using the rich literature of coloration evolution across the animal kingdom to generate and test *a priori* predictions (table 1: A,B), we ask whether specific colours evolve in association with specific patterns in caterpillars. *Hypothesis I: as found in a broad range of taxa, we predict that conspicuous, non-vegetative colours will be evolutionarily correlated with regular, repeating patterns such as bands or spots, and camouflage colours will be evolutionarily associated with patterns such as blotches, stipples, or stripes*.

Next, we ask whether individual colours, patterns or entire colour-pattern ‘syndromes’ evolve in association with ecological traits, drawing from plant defence, plant–herbivore coevolution and predator search theory.

*Hypothesis II: conspicuous coloration may be shaped by the nature of plant chemical defences which, in turn, are associated with plant growth form.* Among prey organisms, toxicity through chemical defence is a key factor shaping coloration strategy, as unpalatability is better learned by predators through conspicuous visual signals [52]. Many species of herbivorous insects gain protection from secondary metabolites within their host plants by either sequestering compounds in their tissues, vomiting repellant regurgitant, or by simply having distasteful plant material in their guts [35,53]: thus, we predict that conspicuous coloration strategies should be linked to chemical characteristics of host plants. More specifically, plant defence theory predicts that herbaceous, non-grass plant species (hereafter forbs) are more commonly defended by acutely toxic chemicals, such as alkaloids and other nitrogen-based compounds than are woody species [54,55], which use large, carbon-rich compounds [54] or grasses, which are primarily defended with silica [56]. Thus, while virtually all plants contain chemical defences, those in some plants may be more effectively co-opted than others: indeed, the compound classes commonly found in sequestering species of Lepidoptera—alkaloids, cyanogenic glycosides, aristolochic acids and others [35]—tend to be more common in non-woody plant species [54]. Following this logic, we predict that conspicuous coloration should evolve with forb-feeding in larval Lepidoptera, while camouflage should predominate in species feeding on woody hosts or grasses.

*Hypothesis III: greater architectural complexity of host plants should favour hiding coloration.* The visual search environment in which prey are found can also determine efficacy of coloration strategy. Complex signal environments may favour camouflage because prey are difficult to detect, making hiding a better defence than being apparent [37]. By contrast, in simpler or more exposed environments where prey are unavoidably visible, selection may favour conspicuous coloration [4,5,39]. Because host plants provide key visual context for predators of herbivorous insects, architectural complexity can provide an alternative hypothesis for the evolution of protective coloration: plants with narrow leaves or smaller stature—such as grasses and herbaceous plants—may favour the evolution of conspicuous coloration [5], while camouflage strategies may be more effective within larger, more architecturally complex woody trees and shrubs. Predictions can be extended to feeding behaviour within host plants: herbivores found in highly exposed locations—such as on plant reproductive parts—may benefit from conspicuous coloration [39]; by contrast, species consuming plant tissues hidden from predator view, such as those that bore within roots or stems, may capitalize on camouflage [44].

*Hypothesis IV: dietary specialization and generalization may favour distinct coloration syndromes.* The evolution of coloration strategy may also be associated with breadth of an organism's niche. Conspicuous coloration could liberate organisms from the constraint of matching specific visual backgrounds, enabling broader resource use [4]. Alternatively, adaptations that enable consumption of toxic diets may trade-off with ability to use other resources, resulting in conspicuously coloured specialists [31,40]. Indeed, recent reviews find that specialist insect herbivores are more likely than generalists to derive chemical defences from host plants [53]. On the other hand, camouflage coloration might also evolve in conjunction with dietary specialization, as strategies that reduce predator detection often require precise mimicry of background elements [57]. Despite the centrality of specialization to theory of coloration evolution, the link between diet breadth and protective coloration, independent of sexual signalling [19,31] has yet to be addressed in a large-scale comparative framework (but see [5]).

To explore these classic and contemporary hypotheses of forces shaping protective coloration, we tested for macroevolutionary associations between colour and pattern, host plant growth form, and dietary specialization in 1808 North American caterpillar species.

## 2. Methods

We used field guides to collect larval coloration data for 1808 species of North American macrolepidoptera across 21 families (electronic supplementary material, table S12). Reflecting North American and global patterns of richness, the families Noctuidae, Geometridae and Erebidae had highest representation in our dataset (electronic supplementary material, table S6). Like many insects, caterpillars undergo remarkable morphological transformations across ontogeny [58]; to account for this, only images of mature (fifth instar) larvae were scored, following instar descriptions in field guides. We chose to focus on late-instar larvae because larger body sizes are favoured by bird predators [59] and send stronger visual signals [60]; thus, this developmental stage may experience particularly strong selection for protective coloration [58,59]. For polymorphic species, we selected an image of one morph at random. Each photograph was independently examined by two observers.

### (a) Coloration

Observers recorded presence/absence of five patterns: stripe; band, spot, stipple and blotch (figure 1) (see also [10]), as well as the three most salient colours to the observer using 11 basic colour categories: brown, green, white, red, orange, yellow, black, grey, pink, blue and purple [61] (see also [10]). We focused on colour and pattern present on the caterpillar body, as head capsules are inconsistently shown in field guides. Photographs were uncalibrated and taken under variable light conditions and therefore not suitable for spectral or pixel analysis [10,62]; under these circumstances, human visual scans are advantageous as they can adjust for illumination differences [10,63]. This approach may fail to detect some visual signals used by birds, which have greater colour discrimination and can see into the UV range; however, birds often detect prey phenotypes similarly to human subjects ([64,65], but see [66]).

Colour combinations can send signals distinct from individual component colours: for example, yellow may serve a cryptic function when occurring with green [28], but a warning function with black [9,24]. To disentangle whether certain colour combinations are associated with pattern and dietary ecology, we also created composite variables of two-colour combinations; for example, *Schinia gaurae* (figure 1) would be coded as having six colour traits: yellow, black, white, yellow/black, black/white and yellow/white. To reduce spurious correlations and number of tests, we excluded rare colour traits with fewer than five occurrences (*n* = 9 traits) as well three-colour combinations, resulting in 49 colour traits (11 single colours + 38 two-colour combinations) for analyses. Including triple, dual and single colours resulted in too many factor levels (*n* = 120) to meaningfully interpret. Following discussions in the literature, we refer to black, and black in combination with yellow, orange, and red as ‘conspicuous’ coloration [67,68]. Colours characteristic of vegetation backgrounds (brown, green and grey, their combinations, and in combination with yellow or white) are considered ‘camouflage’ [10,28,67,69]. We also use these categorizations to inform *a priori* and exploratory model subsets (see the electronic supplementary material, Methods).

### (b) Ecological traits

We used the same field guides to categorize each species as monophagous (consuming a single plant genus), oligophagous (single plant family) and polyphagous (multiple families) [70] (see the electronic supplementary material for details). We also recorded the primary plant tissue type consumed: reproductive tissue (flowers, fruits or seeds); interior tissue (stems or roots); leaves or other (detritus/dead leaves, lichen or fungi). We used the USDA PLANTS database (https://plants.sc.egov.usda.gov) to categorize the growth form of host plant(s) consumed as forb (including geophytes), graminoid or woody shrub/tree.

### (c) Comparative methods

To place our dataset into an evolutionary framework, we estimated a maximum-likelihood phylogeny of study species using nine loci in the National Center for Biotechnology Information's genbank, with a family-level topological constraint tree from Kawahara *et al*. [71] (see the electronic supplementary material, Methods and tables S7–S11). We used phylogenetic logistic regression following the approach of Ives & Garland [72] (function ‘phyloglm’, method = ‘logistic_IG10’; R package *phylolm*) [73] to detect associations between colour, pattern, host and diet traits. Each trait could take a 1 (present) or 0 (absent) for each species (see the electronic supplementary material for model structures). We penalized significance for multiple tests (function ‘p.adjust’, method = ‘fdr’, R package *stats*) [74] within exploratory and *a priori* (e.g. derived from the literature) model subsets, considering both positive and negative correlations (see the electronic supplementary material for details) [75]. To account for observer subjectivity in colour and pattern assignment, we repeated analyses on 1000 bootstrapped trait datasets in which we randomly selected colour and pattern traits from one observer per species. We required that associations be significant in 90% of bootstraps (see the electronic supplementary material, tables S4 and S5).

## 3. Results

We find striking correlations between protective colour and pattern, and among colour, pattern and diet ecology that emerge repeatedly across caterpillar clades. We discuss the most salient results below. See electronic supplementary material for full list of result correlations and discussion, and electronic supplementary material, table S1 for trait frequencies.

### (a) Hypothesis I—associations between colours and patterns

**Figure 2.**
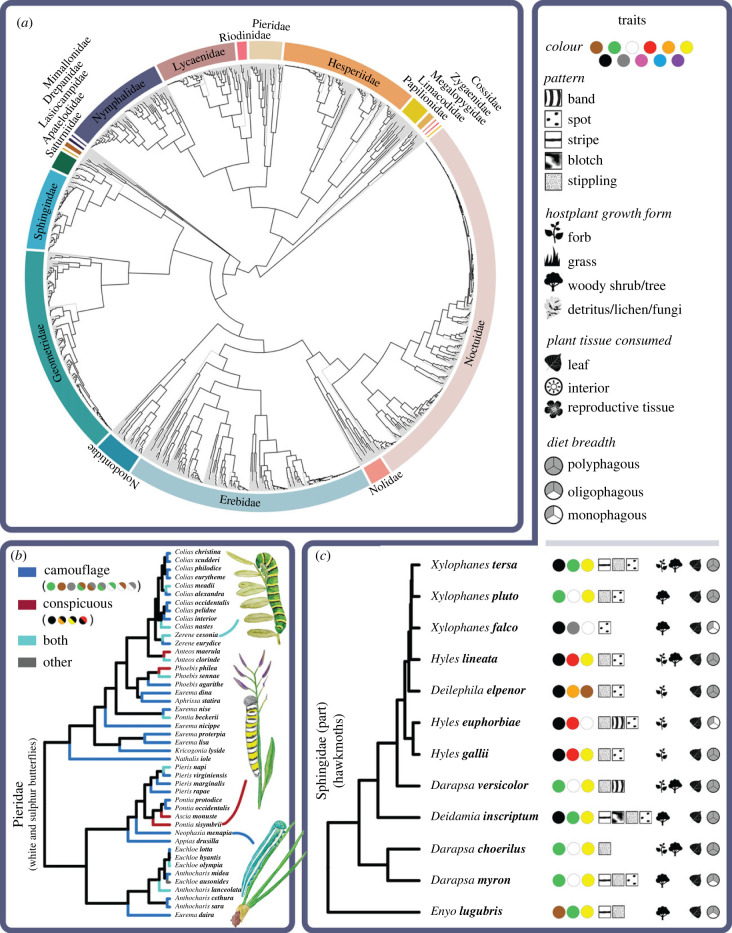
Repeated evolution of protective coloration strategy, and associations between colour, pattern and diet ecology in larval butterflies and moths. (*a*) Phylogeny of 1808 North American Lepidoptera species in this study. (*b*) Colours and colour combinations considered conspicuous and camouflage (see Methods) evolve repeatedly in larval Lepidoptera, shown here for the Pieridae. Tips indicate species containing camouflage colours (e.g. *Neophasia menapia,* green/white: bottom illustration); conspicuous colours (e.g. *Pontia sisymbrii,* yellow/black and black/white: middle); both (e.g. *Zerene cesonia*, green and yellow/black: top); or other (*Euchloe ausonides*, yellow/purple/white: not illustrated)*.* Conspicuous and camouflage colour categories are used to test hypotheses from the literature, while ‘other’ colours are used in exploratory model subsets (see Methods; electronic supplementary material, table S2). (*c*) Evolutionary associations between colour, pattern and diet ecology are shown for a subclade of Sphingidae, highlighting the joint evolution of conspicuous colours with forb-feeding (*Hyles euphorbiae, Hyles gallii*) and camouflage colours with woody plant hosts (*Enyo lugubris, Xylophanes pluto*). All significant associations between colour, pattern and trophic ecology are shown in figure 3.

**Figure 3.**
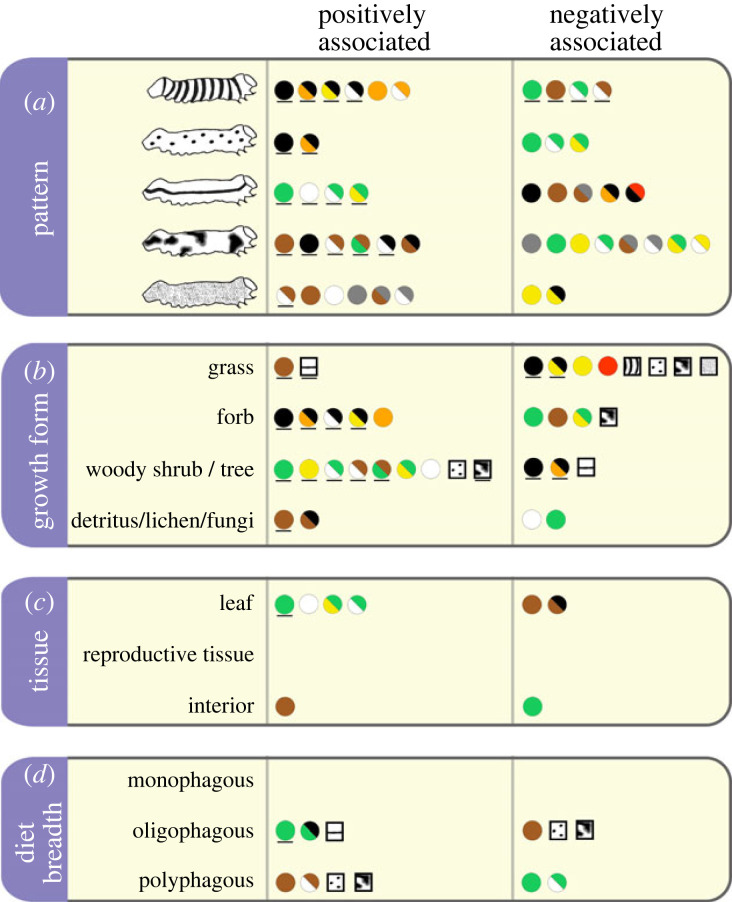
Summary of significant associations between conspicuous and camouflage colours, patterns and dietary ecology. Circles show single-colour or two-colour combination traits; squares represent pattern traits. (*a*) Associations between pattern and colour, corresponding to results from hypothesis I; (*b*,*c*) associations between host plant characteristics and protective coloration (hypotheses II and III); and (*d*) significant associations between caterpillar diet breadth and protective coloration (hypothesis IV). Significance is calculated as the proportion of 1000 bootstrapped datasets yielding an association at *p* < 0.05 or less, after false discovery rate adjustment for multiple tests. See the electronic supplementary material, tables S4 and S5 for effect sizes and significance thresholds. Underline identifies associations predicted from theory (*a priori*).

We find correlations between patterns, colours and colour combinations that suggest distinct syndromes to warn or hide in caterpillars, and that emerge repeatedly across the Lepidopteran phylogeny (figures 2*b* and 3*a*). Banding is positively associated with conspicuous coloration of black in combination with yellow, orange and white (figure 3*a*; electronic supplementary material, table S4) and negatively associated with camouflage colours of green, brown and their combination with white. By contrast, longitudinal stripes are associated with green, white and green/yellow, and negatively associated with conspicuous combinations of orange/black and red/black (figure 3*a*). Similar to bands, spots are associated with black and orange, and negatively associated with green in combination with white and yellow. Irregular blotches are found with brown and black in combination with white—but tend not to be found with other camouflage colours of green, yellow, grey and their combinations with white. Stippling is associated with brown, white, grey and their combinations, and unlikely to be found with conspicuous colours of yellow and black (figure 3*a*; electronic supplementary material, table S4).

### (b) Hypotheses II and III—associations between coloration, host plant growth form and tissue consumed

Consistent with expectations from plant defence theory and host plant-derived toxicity, caterpillars with conspicuous coloration—single colours orange and black, and combinations orange/black, white/black and yellow/black—are likely to consume forbs (figures 2*c* and 3*b*). Species containing black, and combinations orange/black or yellow/black are also unlikely to feed on woody hosts or grasses (figure 3*b*; electronic supplementary material, table S5). By contrast, feeding on woody hosts is evolutionarily correlated with irregular blotches and with vegetation colours (green, brown, white, yellow and their combinations) (figures 2*c* and 3*b*), and feeding on grass is associated with longitudinal stripes and brown (figure 3*b*). Caterpillars with bands were unlikely to eat grass, and more likely to eat forbs (raw significance only; see the electronic supplementary material, table S5). Feeding on flowers, fruits or seeds is not associated with any pattern or colour, while feeding on leaves or interior tissues is associated with vegetation colours (figure 3*c*).

### (c) Hypothesis IV—coloration and dietary specialization

We find no relationship between diet specialization (monophagy or oligophagy) and conspicuous coloration (figure 3*d*; electronic supplementary material, table S5), despite predictions [31,40]. Instead, both family-level specialists (oligophagous species) and broad generalists (polyphagous species) show camouflage coloration, although with mutually exclusive camouflage colours and patterns: oligophagous species are likely to contain green and be striped, and unlikely to contain brown, blotches or spots; by contrast, polyphagous species are likely to contain brown, blotches and spots, and unlikely to contain green (figure 3*d*).

## 4. Discussion

In one of the largest macroevolutionary studies of animal coloration to date (see also [76–78]), our findings corroborate early ideas of why the divergent strategies of conspicuousness and camouflage might evolve, and the role of tri-trophic interactions in the evolution of caterpillar coloration. Our macroevolutionary analyses reveal four main findings: (i) conspicuous and camouflage colours evolve with specific patterns, largely paralleling protective coloration strategies found across a diverse array of taxa; (ii) specific colour and pattern traits within these ‘syndromes’ evolve in association with dietary ecology: both caterpillar colour and pattern are associated with host plant growth form, consistent with predictions based on plant defensive chemistry and architecture; (iii) feeding site is associated with camouflage colours, but not specific patterns; and (iv) broader diets are associated with both camouflage colour and pattern while, counter to many predictions, specialization is not associated with conspicuous coloration elements. We elaborate each of these below.

### (a) Joint evolution of colour and pattern in caterpillars suggests syndromes to warn or hide

We find that caterpillar colours and patterns evolve in specific combinations with each other, suggesting distinct ‘syndromes’ consistent with those in other taxa to confer concealment or conspicuousness (hypothesis I). Conspicuous colours are more likely to be found with high contrast, repeating pattern elements (bands and spots), while camouflage colours are found in association with patterns that blend with background shapes (blotches and stripes) or reduce dimensionality (stipples). Surprisingly, stripes are much more likely to occur with vegetation colours, possibly functioning as disruptive coloration along linear petioles, grass leaves or twigs [12,14,15], and suggesting a more exclusively camouflaging role for this pattern element than found in previous studies [23]. We also find that colour combinations may be more important to signal function than individual colours [9,68]: for example, black is found with yellow and orange in banded species, and with brown in blotched species (figure 3).

In addition to revealing key coloration syndromes among larval Lepidoptera, our findings lend support to common protective coloration strategies across the animal kingdom, despite differences in caterpillar body size and ecology. For example, conspicuous colours black, yellow and orange evolve with transverse bands and regular spots in caterpillars—an association that has also been found in frogs, snakes, lanternflies and other species (table 1; electronic supplementary material, S2). In particularly intriguing similarity, caterpillars and snakes share conspicuous coloration conferred by black associated with orange, yellow and white, as well as background-matching coloration of brown with irregular blotchy patterning [12,20]. Together with previous work finding mimicry of serpentine eyes and posture by Lepidopteran caterpillars and pupae [79,80], these results may point to additional axes of signal convergence between snakes and caterpillars, perhaps owing to shared selective pressures imposed by visual predators [17,20]. Interestingly, while black/red is a conspicuous combination found in many taxa (table 1; electronic supplementary material, S2), it is not common in caterpillars—possibly because foliage often has red components [81] and would not provide contrast.

Caterpillar coloration to reduce detection also shares similarities with diverse taxonomic groups: colours associated with longitudinal stripes in caterpillars (green, white and yellow) are thought to confer crypsis in *Timema* stick insects [30] and chameleons [82]—and were anecdotally suggested to obscure caterpillars against their host plants [15,28]. In the first large-scale investigation of stippling, we find this pattern type in association with camouflage colours, aligning with early natural history notes proposing that these tiny dots could provide a mode of countershading, ‘flattening out’ a prey organism to make it more leaf-like [16]. Stippling provides crypsis in cuttlefish [83], and stippled morphs of golden frogs rest on inconspicuous dead leaves unlike their brightly coloured counterparts [84]. We also find that black in combination with white is associated with both camouflage (blotch) and conspicuous (band) patterns—a dual role also hypothesized in mammals [85].

### (b) Coloration strategy reflects plant growth form and plant defence theory

Our results suggest that host plant growth form shapes distinct evolutionary outcomes for herbivore protective coloration. We find that vegetation colours, blotches and spots are more commonly found in caterpillars feeding on woody hosts (hypothesis II). Spots or blotchy patterns are thought to confer crypsis in the dappled-light understory habitats used by mammals [11], reptiles [1,10] and even plants [13], suggesting that these pattern elements may confer camouflage across diverse taxa that occupy wooded habitats. By contrast, conspicuous, warningly coloured caterpillars are evolutionarily correlated with forb-feeding (hypothesis II), a result consistent with herbaceous plants producing the types of defensive compounds that are readily sequesterable by caterpillars, or that otherwise confer greater toxicity or unpalatability [35,36,54]. Exceptions to the growth form associations in our data also point to a critical role for plant chemistry: among woody plant-feeding *Zale* (Noctuidae), the orange, black and white *Zale perculta* is uniquely conspicuous among its cryptic congeners [86] and feeds on an Ericaceous shrub containing highly toxic grayanoid diterpenes [35]. Thus, our results suggest a critical role for phytochemistry in shaping herbivore protective coloration.

For insects on plants, the smaller stature and less complex architecture of forbs and grasses, or of highly exposed tissues within host plants, were also hypothesized to favour greater conspicuousness [5,39] (hypothesis III). However, we find repeated evolution of cryptic coloration in grass-feeding caterpillars—longitudinal stripes with camouflage colours (e.g. *Mocis latipes*, Erebidae; *Satyrodes eurydice*, Nymphalidae; *Ochlodes agricola*, Hesperiidae)—contrasting with expectations of these habitat complexity hypotheses [5]. Instead, this finding is more consistent with the idea that plant chemical defences shape caterpillar coloration: grass-feeding species are unlikely to gain toxic compounds from their hosts [35,87], and selection has favoured coloration to obscure their form against host plant backgrounds. The association between longitudinal stripes and grass-feeding represents, to our knowledge, the first large-scale finding of a link between grassy habitats and stripe evolution, an idea predicted by early naturalists ([27] with respect to striping in tigers, and of bitterns in reeds, but see [11]), and is consistent with a disruptive or background-matching role for striping in taxa occupying linear backgrounds [1,14,82].

We also found that caterpillar pattern was unrelated to the tissue type they consume, suggesting that many pattern elements can confer protection across leaf, reproductive tissue and interior tissue diets. Instead, colour is more closely associated with leaf and interior-feeding, with prevalence of green (leaf) and brown (interior) indicating that camouflage is a common strategy in species consuming these tissue types. Despite theoretical predictions, we fail to link conspicuous coloration with flower and fruit-feeding [39] (hypothesis III), suggesting that florivores may use behavioural adaptations or refugia—such hiding within developing fruit capsules (e.g. *Hadena ectypa* (Noctuidae) [86])—to avoid predation. Alternatively, species with unavoidably exposed larvae may evolve divergent strategies to either stand out or precisely match plant reproductive structures (electronic supplementary material, figure S1).

### (c) Both broad and narrower diets may favour camouflage coloration

Diet specialization can result in highly efficient co-opting of toxins [31,88] and is often advertised by conspicuous coloration [31,40] (hypothesis IV). However, we find no relationship between host plant specialization (monophagy and oligophagy) and the evolution of conspicuous coloration (see also [58]). We suggest that, for the most specialized species (monophagous; one plant genus), both conspicuous and camouflage strategies may be effective when diets—and thus visual backgrounds—are highly specific [58,89]. There may also be interactions with host plant growth form: specialists on forbs might be conspicuous, owing to availability and efficient use of toxins in their diets [54], while specialists on woody hosts lack this source of chemical protection and must rely on camouflage.

While the most highly specialized species (monophages) do not use any particular coloration strategy, both family-level specialists (oligophagous species) and generalists consuming multiple plant families (polyphagous species) are likely to be camouflaged. A possible explanation is that, although chemical compound classes tend to be similar within plant families [90,91], even closely related plants can confer different levels of protection to their consumers [92]. Thus, co-opting plant chemicals may be less effective when diets span multiple plant genera or families [88], favouring strategies to blend in rather than stand out. We also find that oligophagous species are likely to be green, not brown, and have longitudinal stripes, suggesting a camouflage strategy against green leaf or stem backgrounds; while polyphagous species contain blotch and spot pattern elements, in combination with brown. Possibly, a very broad host plant diet may increase likelihood of travel within and between plant individuals [93], thus favouring brown coloration as a compromise strategy among heterogeneous microhabitats [94] or to blend in against soil or wood during bouts of high-risk movement [95]. Diet generalization may also be associated with behavioural adaptations such as nocturnal feeding and resting on bark or dead leaves away from host plant foliage [96], which would favour brown coloration ([42], but see [97]). Together, these results are contrary to the hypothesis that conspicuous coloration promotes broader niche breadth [98] and are consistent with the idea that generalists are poorer at co-opting plant defences [35,88].

There are several caveats to consider when interpreting our results. First, additional traits such as spines, hairs or communal behaviour may influence perception and function of protective coloration [99]. Second, our study may overlook polymorphic species that employ multiple strategies to avoid attack—though our random image selection approach should avoid bias. Third, we may not expect the same divergence in caterpillar coloration strategy between herbaceous and woody hosts in tropical systems, as woody plants in the tropics often contain acutely toxic compounds (e.g. [100]). Coloration of grass-feeding species may be more globally consistent, however, as recent studies find weaker latitudinal clines in traits of grasses [101]. Despite these considerations (see the electronic supplementary material for further discussion), we find overarching associations between pattern, colour and diet ecology that are robust across many families of both caterpillars and host plants, and consistent with strategies to avoid attack in many other taxa.

In conclusion, we use one of the most diverse insect orders to relate macroevolution of protective coloration to trophic ecology and support long-held ideas of plant–insect coevolution. We find repeated, joint evolution of colours and patterns to confer conspicuousness or camouflage, and identify specific coloration elements that may be key to lepidopteran success across diverse host plants. Our results point to tri-trophic roles of predators and plant chemistry and, less so, plant architecture, in shaping herbivore coloration. These results link classic hypotheses of plant defence theory and toxicity to evolution of protective coloration strategy. By testing foundational ideas of signal function and finding new associations in a key group of terrestrial prey organisms, we broaden our ecological understanding of coloration evolution across the animal kingdom and reinforce the critical role played by pattern and colour to hide and warn.

## Data Availability

Our paper presents new data. Data are available from Figshare: https://doi.org/10.6084/m9.figshare.19435760.v3 [102]. Code is available here: https://github.com/moria-robinson/macroevolution-of-caterpillar-coloration. Data are also provided in the electronic supplementary material [103].
